# Thin-Film-Based Multifunctional System for Optical Detection and Thermal Treatment of Biological Samples

**DOI:** 10.3390/bios12110969

**Published:** 2022-11-04

**Authors:** Nicola Lovecchio, Francesca Costantini, Augusto Nascetti, Giampiero de Cesare, Domenico Caputo

**Affiliations:** 1 Department of Information Engineering, Electronics and Telecommunications, Sapienza University of Rome, 00184 Rome, Italy; 2 CREA-DC Research Centre for Plant Protection and Certification, 00156 Rome, Italy; 3 School of Aerospace Engineering, Sapienza University of Rome, 00138 Rome, Italy

**Keywords:** Lab-on-Chip, amorphous silicon sensors, luminescence detection, fluorescence detection, temperature control system, portable multifunctional platform, DNA amplification system

## Abstract

In this work, we present a multifunctional Lab-on-Chip (LoC) platform based on hydrogenated amorphous silicon sensors suitable for a wide range of application in the fields of biochemical and food quality control analysis. The proposed system includes a LoC fabricated on a 5 cm × 5 cm glass substrate and a set of electronic boards for controlling the LoC functionalities. The presented Lab-on-Chip comprises light and temperature sensors, a thin film resistor acting as a heating source, and an optional thin film interferential filter suitable for fluorescence analysis. The developed electronics allows to control the thin film heater, a light source for fluorescence and absorption measurements, and the photosensors to acquire luminescent signals. All these modules are enclosed in a black metal box ensuring the portability of the whole platform. System performances have been evaluated in terms of sensor optical performances and thermal control achievements. For optical sensors, we have found a minimum number of detectable photons of 8 × 10^4^ s^−1^·cm^−2^ at room temperature, 1.6 × 10^6^ s^−1^·cm^−2^ in presence of fluorescence excitation source, and 2.4 × 10^6^ s^−1^·cm^−2^ at 90 °C. From a thermal management point of view, we have obtained heating and cooling rates both equal to 2.2 °C/s, and a temperature sensor sensitivity of about 3 mV/°C even in presence of light. The achieved performances demonstrate the possibility to simultaneously use all integrated sensors and actuators, making promising the presented platform for a wide range of application fields.

## 1. Introduction

Portability, quick response time, and low reagents consumption are just some of the Lab-on-Chip (LoC) advantages that have led this technology to be among the major research focus of many academic and industrial groups over the last two decades [1,2,3,4,5]. The applications that can be developed by using LoC-based systems cover several research fields, starting from environment monitoring [6,7,8] toward health care and biomedical applications for disease detection and treatment [9,10,11,12,13].

Among these fields, the concept of “true” Lab-on-Chip began to catch on more and more, highlighting increasingly differences between real portable devices and those that can be defined Chip-on-a-Lab due to the bulky external components they need [14,15,16,17]. For this reason, integration has become the key focus of these kinds of systems, and a lot of research works can be found in the literature on how to integrate and miniaturize sensors and actuators [18,19,20,21].

Looking at the integration possibilities, hydrogenated amorphous silicon (a-Si:H) is an appealing material, since it is suitable for developing both active and passive devices on a variety of substrates and on large areas [22,23,24,25]. Thanks to the low thermal budget required by the Plasma Enhanced Chemical Vapor Deposition (PECVD) technique [26,27,28], the a-Si:H can be deposited on silicon, glass, metals, several plastic substrates, and so forth, allowing therefore the integration and miniaturization levels that a “true” Lab-on-Chip requires [29,30].

The possible applications of amorphous silicon-based devices range from multilayer color detectors [31] to active matrices for liquid-crystal displays [32], from thin-film photovoltaic cells [33] to one-, two-, or three-dimensional optical position detectors [34], and its wide use is due both to the intrinsic properties of the material and the mature fabrication technologies. Recently, a-Si:H has been used also for developing light and temperature sensors in Lab-on-Chip applications [35,36,37,38,39], demonstrating the great potential this material has to offer.

In this work, we present a portable LoC-based multifunctional system that, by exploiting the abovementioned properties of the amorphous silicon, allows its use in all applications where the optical detection in the visible range can be used for the detection and quantification of the target analyte. In particular, in Section 2, we report on the structure and operation of the system, describing in detail the design and fabrication process of each module. In Section 3, we evaluate the platform’s intrinsic performances in the framework of feasible applications of the system, without considering the effect of specific samples under analysis. Finally, in Section 4, we summarize the obtained results.

## 2. System Structure and Operation

**Figure 1:**
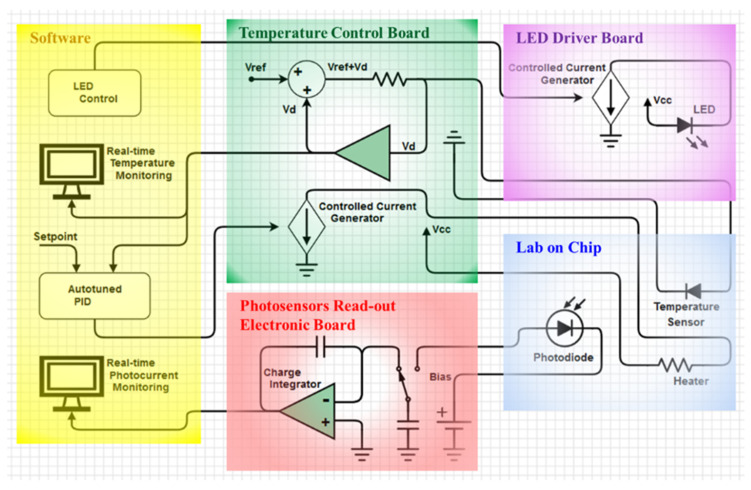
Schematic block diagram of the system structure.

A schematic block diagram showing all the modules of the system is depicted in Figure 1.

The core of the system is the Lab-on-Chip, also called System-on-Glass (SoG), which is a 5 cm × 5 cm glass substrate hosting a thin film heater, a-Si:H light and temperature sensors, and an optional thin film interferential filter suitable for all the applications requiring the fluorescence detection. Thanks to a low-noise charge integrator-based read-out electronics (red block in Figure 1), the optical detection in the visible spectrum range can be performed, including fluorescence-based applications that need an excitation source (violet block). Simultaneously, it is possible to apply thermal treatments on the target analyte thanks to the temperature control board (green block), which infers the temperature by the a-Si:H temperature sensors and drives the thin-film heater to reach the desired temperature. Finally, a custom graphical user interface (yellow block), developed in Visual C++, allows the users to control the complete system.

**Figure 2:**
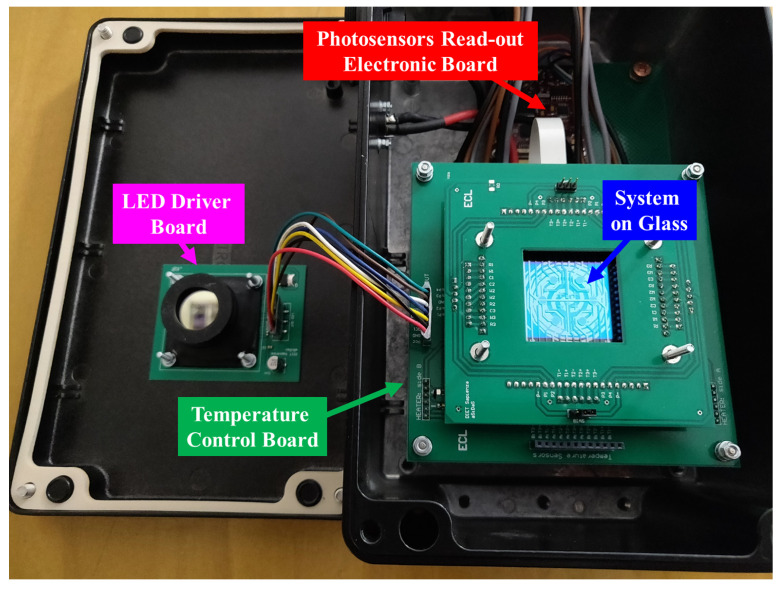
Black metallic box containing the complete system.

All these modules, including the LoC with an ad hoc connector for electrical contacting sensors and heater, are enclosed in a black metallic box whose dimensions are 26 cm × 16 cm × 12 cm, as shown in Figure 2. The total weight of the metallic box is below 2 kg.

A detailed description of the SoG and an explanation on how each module works are reported in the following Subsections.

### 2.1. System-on-Glass

**Figure 3:**
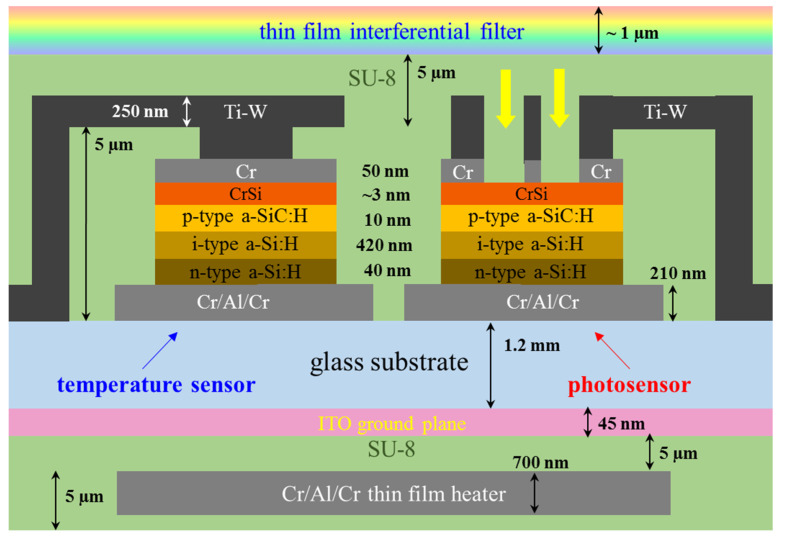
Schematic cross-section of the System-on-Glass.

Figure 3 shows the schematic cross-section of the LoC.

**Figure 4:**
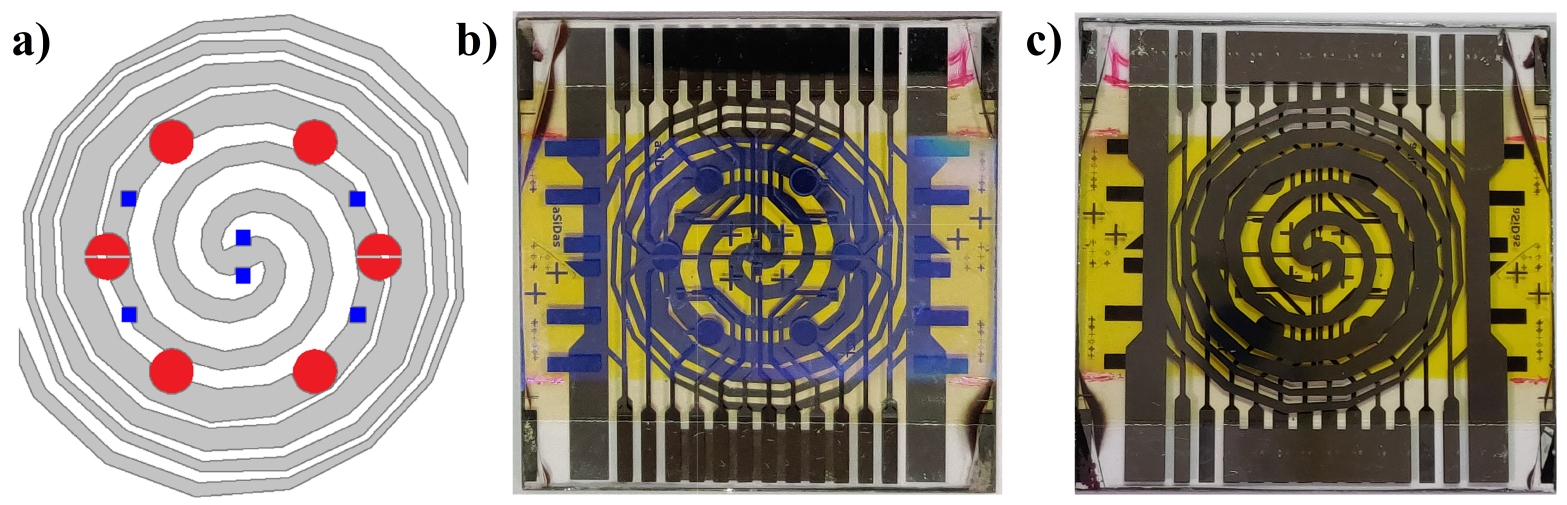
(**a**) Geometry of the thin film heater (gray lines) overlapped by red circles, that indicate the photosensors, and by blue squares, representing the temperature sensors. (**b**) SoG top side, hosting photosensors, temperature sensors, and interferential filter (light brown rectangular band). (**c**) SoG bottom side, hosting the thin film heater.

The LoC is fabricated on a BOROFLOAT^®^ 33 glass substrate from Schott, United Kingdom, which is a high-quality borosilicate glass with good physical properties including high transmission in the visible and infrared light ranges, operating temperature up to 450 °C, resistance against strong acids, bases, and organic substances, low coefficient of thermal expansion, and so forth. All sensors and actuators mentioned above have been integrated on both sides of the substrate, as shown in Figure 4, where a picture of the fabricated System-on-Glass, together with a schematic representation of the heater and the sensors geometries, is reported.

The heater, fabricated on the bottom side of the proposed SoG, is a double concentric spiral-shaped resistor, designed and optimized in terms of pitch and line width by using COMSOL Multiphysics® in order to achieve a uniform temperature distribution, with a temperature variation below 1.5 °C over an about 5 cm^2^ area and up to 100 °C. A detailed description of this heater in terms of static and dynamic response to heating can be found in [40]. On the top side of the SoG, eight photosensors (four round-shaped with a 3 mm diameter, corresponding to a 7 mm^2^ area, and four with semirounded shape with a 3.5 mm^2^ area) have been positioned on a radial distribution obtaining the same dynamic evolution of the temperature during heating. Finally, six square temperature sensors of 1 mm^2^ area have been integrated on the same side: four of them are positioned on the same photosensors radial distribution, while the other ones are placed on the central area.

The presented chip is fabricated according to the technological steps reported below in sequential order:

1. ultrasonic cleaning of the glass substrate;
2. sensors side:
  (a) deposition of a sacrificial protection layer of a 400 nm-thick titanium-tungsten (Ti-W) alloy in a three-targets Material Research Corporation RF magnetron sputtering system;
3. heater side:
  (a) deposition of a 45 nm-thick indium-tin oxide (ITO) layer acting as a ground plane through the same RF magnetron sputtering system;
  (b) deposition of the insulation layer of a 5 µm-thick SU-8 3005 film by spin coating and its patterning to expose the contacts to connect the ground plane;
  (c) deposition, by vacuum evaporation, of a chromium (Cr)/aluminum (Al)/ chromium (30/600/70 nm) stacked layer by means of a Balzers 510 evaporation system and its patterning to define the thin film heater by means of standard optical lithography and wet etching processes;
  (d) definition of a passivation layer through the spin coating of a 5 µm-thick SU-8 3005;
4. sensors side:
  (a) removal of the Ti-W sacrificial protection layer;
  (b) deposition of a Cr/Al/Cr (30/150/30 nm) stacked layer and its patterning to define the sensors bottom contacts;
  (c) deposition of the a-Si:H structure by means of a Glasstech Solar Incorporation three-chamber ultra-high vacuum PECVD system;
  (d) evaporation of a 50 nm-thick Cr layer, acting as the sensors top contact;
  (e) Cr wet etching and a-Si:H dry etching in Ionvac Reactive Ion Etching (RIE) system for the mesa patterning of the diodes;
  (f) deposition of the insulation layer by spin coating of a 5 µm-thick SU-8 3005 film and its patterning for the opening of the via holes;
  (g) sensors side: deposition of a 250 nm-thick Ti-W alloy and its patterning for the definition of the top contact and the connection to the pads, located on the glass edge;
  (h) definition of a passivation layer through the spin coating of a 5 µm-thick SU-8 3005 film;
  (i) optional deposition of a multilayer stacked structure of titanium oxide (TiO_2_)/ silicon oxide (SiO_2_) dielectric layers acting as thin film interferential filter.

Thanks to the design optimization of the system, all the integrated sensors and actuators can operate simultaneously without loosing in performances. In particular, temperature sensors and photodiodes are fabricated in the same deposition run; for this reason, the thicknesses of the p-i-n junction layers have been optimized to have both high sensitivity to light and good linearity and stability when the diodes are used as temperature sensors [25]. Furthermore, as it can be noted in the cross-section of Figure 3, only the top contact differs for these kind of sensors. In particular, for the photosensors, it consists in a Cr/Al/Cr metal grid realized over a thin transparent layer of chromium silicide, which is autonomously formed when the chromium is deposited on the n-doped amorphous silicon [41]. This configuration allows the light radiation to reach the intrinsic layer, where the electron-hole pair can be photogenerated. On the other hand, for the temperature sensors, the top contact is a uniform metal layer able to shield the light radiation, which must not affect the temperature measurements. In addition to the different configuration of the top contact, also a transparent ground plane has been integrated for uncoupling the heater leakage currents from the sensors, leaving intact their sensitivity and, therefore, the system limit of detection also when heater and sensors are used at the same time.

For all the applications requiring the fluorescence detection, a thin film interferential filter has also been integrated [28]. The filter allows to transmits only the emission spectrum of the used fluorescence molecules, rejecting, at the same time, the excitation wavelengths. This reduces the photosensor background signal and avoids the read-out electronics saturation.

### 2.2. SoG Connection System

**Figure 5:**
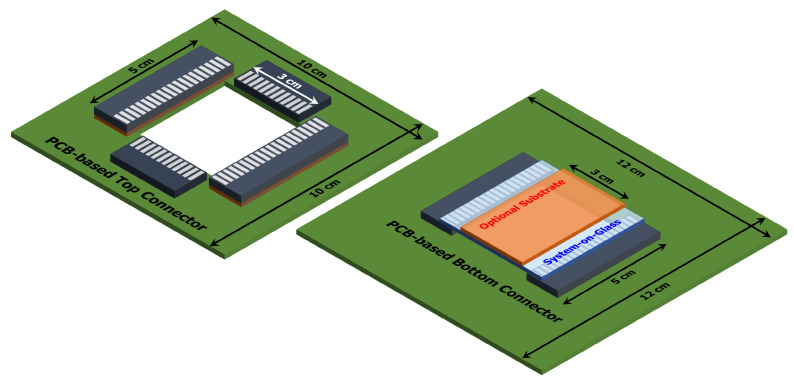
Schematic 3D view of the designed top (**left**) and bottom (**right**) PCBs.

In order to ensure a proper connection between the SoG and the electronics needed to control each module, a custom-made electrical connector based on printed circuit board (PCB) has been developed. A schematic 3D view of the proposed solution is depicted in Figure 5.

The developed connector is composed by two parts, a top and a bottom PCBs. The bottom one integrates two 20-pad connectors for the heater contacts and it is used also to sustain the SoG. On the other hand, the top PCB includes another two 20-pad connectors for the SoG sensors contacts and two supplementary 12-pad connectors suitable to drive optional electrodes. These ones have to be integrated on a 3 cm × 5 cm top substrate, positioned on the top of the LoC as shown in Figure 5. This configuration allows to integrate other sensors and/or actuators to the platform, making feasible the driving, for example, of sensors to monitor conductibility [42,43] or pH [44], electrodes for digital microfluidic [45,46] or electrochemical analysis [47].

The 12- and 20-pad connectors mounted on the PCBs have been obtained by cutting transversely the 5-5530843-4 Edge Card Connector (from TE Connectivity Amp) in order to expose the metallic pads. Moreover, since the substrates can have different heights, some wedges (brown blocks in the figure) are placed under the12-pad connectors in order to correctly contact all of the modules. This kind of connection system ensures both high mechanical stability and strong electrical connection, allowing to reduce contact resistances without damaging the metal thin-film pads of the SoG.

**Figure 6:**
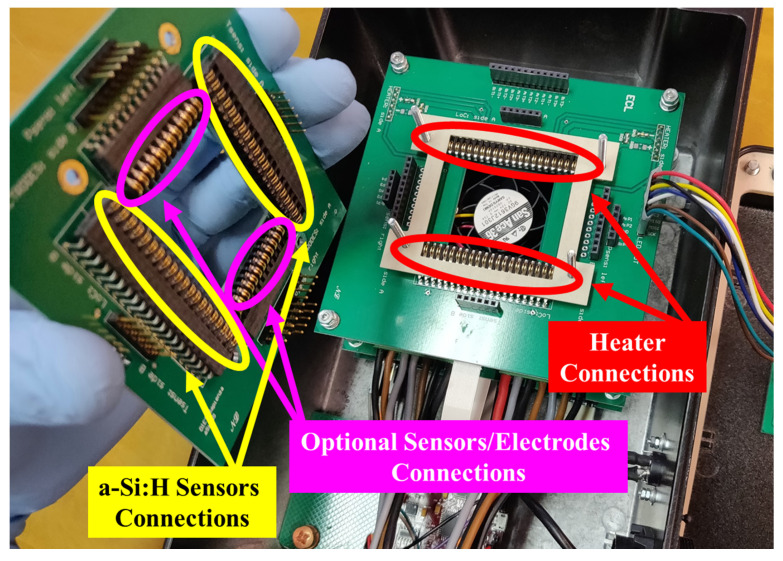
Picture showing the custom-made connector designed to contact heater, sensors, and optional sensors/electrodes.

A picture of the realized custom-made connector is reported in Figure 6.

### 2.3. Thermal Treatment Module

This section describes the temperature control system, including the electronic circuit boards (green block of Figure 1) and the developed proportional–integral–derivative (PID) algorithm [48] to stabilize the temperature. The electronic boards, in turn, include:

- a power circuit that drives the heater with the current required to achieve the desired temperature;
- a power circuit that drives a fan used to enhance the cooling of the LoC if it is required by the specific application;
- an electronic circuit that biases the temperature sensor at a constant current and measures the voltage across the diode junction.

**Figure 7:**
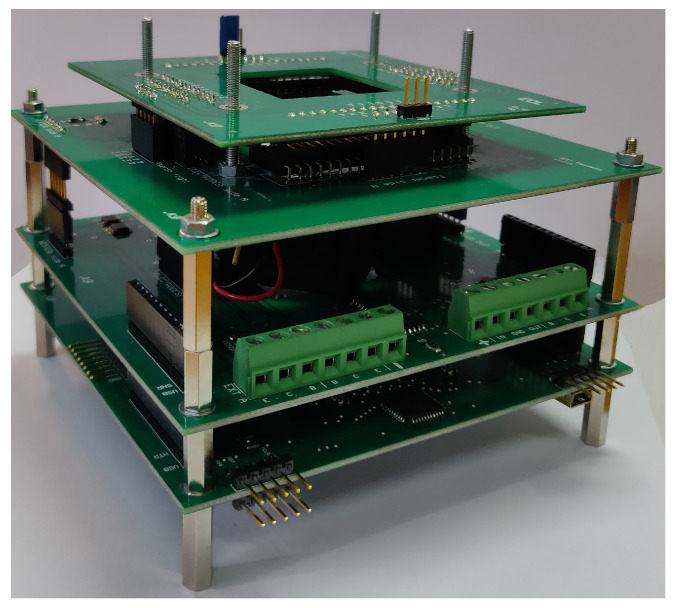
Picture of the thermal control module. The first PCB on the bottom hosts the the temperature acquisition circuit and the microcontroller, while the second one includes the fan, positioned at the center of the board, and the power circuits. As described before, the two PCB on the top are used as connectors for the SoG.

A current generator driven by a digital to analog converter has been used in order to pilot the thin film resistor [49]. Moreover, a fan is placed under the SoG to quickly cool the LoC when the specific application requires thermal cycles or rapid decrease in temperature. The same circuit typology of the heater driver has been used to drive the fan. Both these power circuits have been integrated on the same printed circuit board (PCB), while the circuit that biases the temperature sensors is realized on another PCB (Figure 7). In this way, the electronics employed for the temperature acquisition is uncoupled from the electronic board that hosts the fan and the power circuits, allowing to strongly reduce interferences and, therefore, the noise associated to the temperature detection.

The circuit that infers the heater temperature through the a-Si:H junction (described in [49]) drives a diode with a constant current in forward condition and, at the same time, measures the voltage across the diode. The temperature monitoring is possible since the voltage across the diode used as temperature sensor varies linearly with the temperature when it is biased with a constant current. A detailed characterization of our temperature sensors in terms of sensitivity and stability as a function of the operating point is reported in [25]. Thanks to the electronics optimized design, the minimum detectable voltage signal is about 10 µV that, considering a sensor sensitivity of about −3 mV/°C, corresponds to a temperature value lower than 4 × 10^−3^ °C.

**Figure 8:**
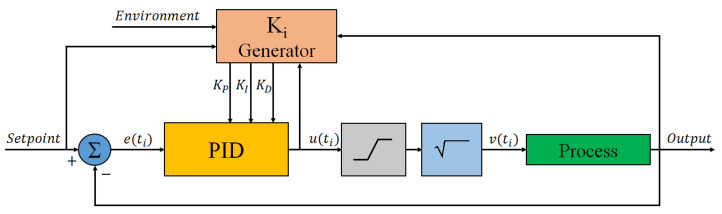
Block diagram of the auto-tuned PID controller in a feedback loop.

A Graphical User Interface (GUI) controls all of the functionalities of the electronic boards. In particular, a software PID auto-tuned algorithm is used to reach a user-specified setpoint temperature starting from the temperature inferred by the diode. The schematic diagram of the developed algorithm is shown in Figure 8.

The block “K_i_ Generator”, which is a real-time PID-parameters estimator implemented as a fuzzy-logic controller [50,51], is used to provide for each cycle the proportional (K_P_), integral (K_I_), and derivative (K_D_) gains to the block “PID”. This block, according to the calculated error and the abovementioned parameters, evaluates a value that is proportional to the power dissipated by the heater.

Since the heater current must be a positive value, a limiter block (gray box in Figure 8) has been introduced to ensure that the PID output is not negative and, moreover, does not exceed the maximum current that can be provided to the heater.

In an ideal thermal system, the temperature reached by the Joule effect is proportional to the dissipated power, which is, in turn, proportional to the square of the current. To linearize the algorithm response, a square root block (light blue box in the figure) has been introduced.

Finally, the block called “Process” represents, in this case, the SoG. The input variable $v(t_{i})$ is the current which flows into the heater, while the variable Output is the voltage across the a-Si:H temperature sensor, and hence the temperature reached by the SoG.

A detailed description of the developed auto-tuned PID algorithm can be found in [52].

### 2.4. Optical Detection Module

The proposed platform exploits optical detection methods, performed by integrated thin film amorphous silicon-based devices, as a sensitive approach for stimulated fluorescence detection, optical absorption measurements, and chemiluminescence or thermochemiluminescence detection. The optical module includes two electronic boards: one (red block in Figure 1) used for the photocurrent acquisition by the light sensors and another one (violet block in Figure 1) used for controlling a light-emitting diode (LED), which is suitable as the excitation source for both absorption measurements and fluorescence detection.

The first PCB is an eight-channel photocurrent readout electronics that combines selection of the current range (from femtoamps to hundreds of nanoamps), good noise performances, and on-board sensor-bias voltage supply [53]. The read-out electronics includes a dual-switched integrator and performs a continuous signal integration with zero dead-time. Thanks to the careful design, the performances of this system have been optimized to work with amorphous silicon photosensors, obtaining a noise level of about 10 fA [53]. Considering that the area of the abovementioned photosensors is 7 mm^2^ and their responsivity is about 350 mA/W [54], the minimum detectable signal of this system is of the order of few pW/cm^2^.

The electronic boards driving the excitation light source include a current generator that controls the LED and a MOSFET-based analog switch, connected in parallel to the LED, to quickly switch-off the excitation source allowing fast blinking operations. On the top of the light source is placed a black 3D-printed holder (visible on the left of Figure 2), in order to lock a collimator and a band-pass filter for directing and shrinking the LED wavelength spectrum, respectively. The board is placed on the box cover and is aligned to the SoG in order to enlighten all the active area, i.e., the 3 cm-diameter round area including all sensors of the LoC. When the box is closed, the distance between the band-pass filter and the SoG is 3 cm.

### 2.5. Microfluidics

**Figure 9:**
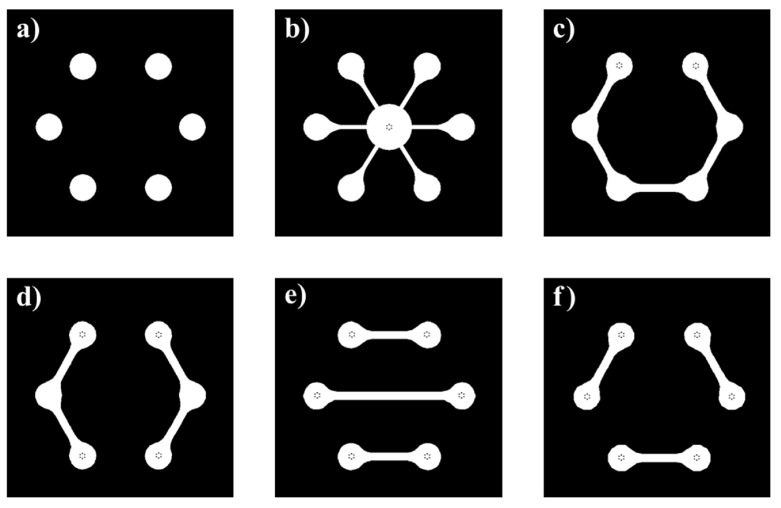
Possible shapes of the microfluidic chip: (**a**) six separated wells; (**b**) configuration including a central load-lock chamber connected to six detection areas; (**c**) microfluidic channel connecting all detection sites; (**d**) two channels configuration; (**e**,**f**) three channel configurations.

In order to exploit the features that the proposed system can offer, an appropriate microfluidic network, which has to be optically and thermally coupled with the SoG [37,38], should be realized. The distribution of the photosensors on the LoC allows different microfluidic geometries, suitable for both stop-flow or in-flow measurements. Some of the possible shapes of microfluidic wells and channels that can be used with the presented system are shown in Figure 9.

**Figure 10:**
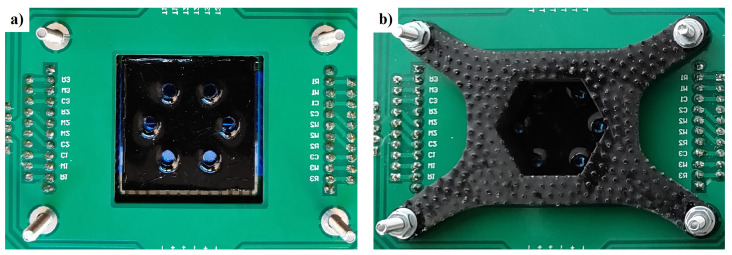
Picture of the microfluidic chip coupled with the SoG without (**a**) and with (**b**) a black 3D-printed holder.

The microfluidic chip can be developed by using different materials, including glass [35], Cyclic Olefin Copolymers (COC) [38], or Polydimethylsiloxane (PDMS) [4]. A picture of the system coupled with a simple black-PDMS six-wells microfluidic chip (Figure 9a) sticked on a 3 cm × 3 cm 200 µm-thick glass substrate is shown in Figure 10, where a 3D-printed holder has been positioned on the top of the SoG to guarantee optical and thermal coupling between the LoC and the microfluidics.

## 3. Evaluation of System Performances

In this Section, an evaluation of the system performances as a function of the possible applications will be presented. The analysis will range from the detection of a simple chemiluminescent biochemical reaction up to the possibility to perform a real-time three-temperatures polymerase chain reaction (PCR), as reported in the next subsections.

It is worth noting that this evaluation is carried out just in terms of a-Si:H sensors and thin film heater performances independently on the biological sample to be detected and the specific diagnostic, agro-food or clinical application. Therefore, all the achieved results do not consider the effects of the real samples, such as the matrix effect for a food quality control, the luminescent efficiency of the fluorophores in a fluorescence-based detection, and so forth. The specific performances will depend on the considered application and are out of the aim of this work. Hence, the presented evaluations characterize the intrinsic performances of the system, as given by the system-on-glass and the control and read-out electronics.

Moreover, all the below reported measurements refer to a stable behavior of the sensors. Indeed, the optoelectronic characterizations performed on “as grown” temperature and light amorphous silicon sensors show that each sensor modifies its characteristics for repeated measurements and thermal cycles. However, these changes decrease with increasing use and, for this reason, all the reported measurements have been carried out after a few days during which the optoelectronic platform is stressed with continuous thermal cycles. After this treatment, the sensors result stable, since the differences between repeated measurements of the same sensor is less than 1% without showing a monotonic trend.

### 3.1. Room-Temperature Luminescence Detection

Several LoC-based applications, as can be found in literature, do not need to treat the analyte [55,56,57] but simply perform the detection by using electrochemical [58,59,60], mechanical [61], or optical methods [21,62,63,64]. Among these, the optical detection has been the most widely used technique for quantitative analysis due to its robustness and sensitivity [2].

**Figure 11:**
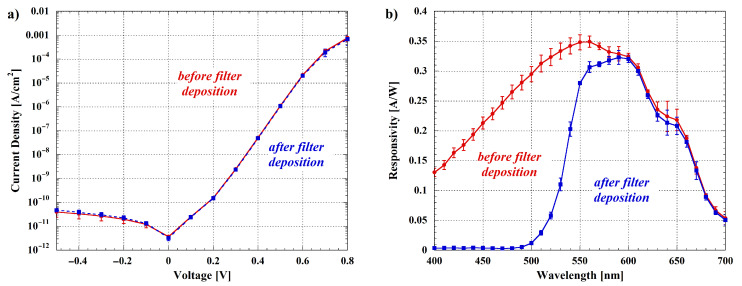
(**a**) Current density–voltage (JV) characteristics and (**b**) responsivity as a function of wavelength of the fabricated photosensors. Curves have been measured before and after interferential filter deposition. Error bars refer to measurements on the eight integrated photosensors for both JV and responsivity graphs, and standard deviations are less than 10^−4^ A/cm^2^ and 0.03 A/W, respectively.

Chemiluminescence (CL) is a widely employed optical method for analyte detection, where the target binding induces a direct or an enzyme-labeled photochemical emission. This technique is particularly attracting because it avoids the presence of an excitation source, minimizing therefore the background signal noise. For LoC systems, the benefits are even greater since the absence of the excitation source and filters improves the system portability [65]. However, highly sensitive detectors are typically demanded. For this reason, hydrogenated amorphous silicon photosensors are appealing for chemiluminescence detection in the visible wavelength range, since low dark current and good responsivity can be obtained, as shown in Figure 11a and in Figure 11b (red curves), respectively.

By biasing the photosensors with a small reverse voltage (below 100 mV), the related dark current density results to be less than 10 pA/cm^2^ with an associated shot current noise around 6 fA/(cm^2^√Hz). Considering that the noise of the read-out electronics is around 10 fA [53] and a limit of detection equal to 3 times the system noise, the minimum detectable photocurrent *I_ph,min_* is close to 50 fA/cm^2^. On the other hand, taking into account that the photon number *n_ph_* can be derived from the following equation:

(1) $$n_{ph}=\frac{I_{ph}\cdot \lambda }{q\cdot R\cdot 1240}$$

where λ is the wavelength, *q* is the electron charge, and *R* is the responsivity, the minimum number of detectable photons in a chemiluminescent signal (*n_ph,min_*) is estimated to be 8 × 10^4^ photons/(s cm^2^). Up to our knowledge, this value greatly improves the detection limit found in literature [66]. Thanks to the obtained performances, the system allows low limit of detections, as recently demonstrated for several applications [67,68,69].

Even if the CL technique is preferable respect to others photoemission mechanisms due to the intrinsic compactness that derives from this method, the fluorescence detection results to be the widely used method for the analyte detection and quantification in lab-scale applications [70]. Fluorescence is a luminescence mechanism caused by the excitation of fluorescent molecules, through the absorption of the energy coming from an incident radiation. The fluorescent agent re-emits almost immediately (within about 10^−8^ s), usually at higher wavelengths than the exciting ones. Since the re-emission occurs so quickly, the fluorescence ceases as soon as the exciting source is removed, making the detection of the analyte possible only in presence of the excitation light.

Despite several possibilities for integrating compact fluorescence sensors on LoC systems have been developed [71], the employment of bulk optical components, like lenses or optical fibers, are still used for the coupling of the detector and the fluorescence source [72]. This external optical coupling strongly reduces the portability of the developed systems. The presented platform overcomes these issues, making possible the fluorescence-based detection thanks to the integrated thin film interferential filter, which rejects the excitation source without increasing the distance between the fluorescence site and the photosensors. However, in order to ensure a proper transmittivity range of the interferential filter, the selected fluorescent molecule should have a high Stoke shift, which is the distance between the absorption and the re-emission wavelengths. In this way, it is possible to reject the excitation light by an accurate design of the filter band, and have at the same time the maximum transmittance at the molecule re-emission wavelength. Ruthenium-based fluorophores are very appealing to this aim, since the absorption of these complexes is in the range between ultraviolet (UV) and blue light, while the re-emission spectra are usually positioned beyond 500 nm [73,74]. In particular, the interferential filter integrated on the presented SoG has been dimensioned, as reported in [28], to work with the [Ru(phen)_2_(dppz)]^2+^ (phen = 1,10-phenanthroline, dppz = dipyrido[3,2-a:2${}^{\prime }$,3${}^{\prime }$-c]phenazine), a fluorescent dye that shows an absorption peak around 450 nm, and an emission peak located between 610 and 630 nm. Experimental results related to the filter operation are shown in Figure 11b, where the blue curve represents the responsivity of the photosensors after the filter deposition. As expected, the responsivity around 450 nm is below 10^−3^ A/W, confirming the ability of the interferential filter to reject the excitation light source without degradating the photosensors performances, as also demonstrated by the blue curve of Figure 11a.

To evaluate the system performances in detecting fluorescent signal, we need to take into account not only the noise associated to the sensor dark current and to the read-out electronics but also the noise associated with the background signal due to the excitation light. In order to find this value, we have made several experiments varying the ruthenium complex concentration in the microfluidic holes and the power intensity of the excitation source. We have found that the best compromise for maximizing the signal-to-noise ratio is achieved when the ruthenium complex concentration ranges between 1 and 5 µM while the driving current of the LED radiation source ranges between 0.3 and 0.5 mA. In these conditions, the minimum photocurrent is around 3 nA/cm^2^, which corresponds to a background radiation shot noise of 40 fA/(cm^2^√Hz). Once again, considering a limit of detection equal to 3 times the system noise, the minimum detectable photocurrent is close to 150 fA/cm^2^. Following the Equation (1), we can estimate that n_ph,min_ at 610 nm is around 1.6 × 10^6^ photons/(s cm^2^).

Recently, authors have demonstrated the suitability of the proposed fluorescence detection system in a quality food control application [67]. In particular, the possibility to detect and quantify the concentration of the Ochratoxin A in beer and wheat was demonstrated, obtaining a limit of detection and a limit of quantification of 1.3 ng/mL and 3.9 ng/mL, respectively.

Electrochemiluminescence (ECL) is another photoemission mechanism that can be exploited for the detection and quantification of the target analyte in LoC-based systems [75,76,77]. It is usually observed during the application of a potential (several volts) to the electrodes of an electrochemical cell that contains a solution of luminescent species (polycyclic aromatic hydrocarbons, metal complexes, Quantum Dots or Nanoparticles) in an aprotic organic solvent [78]. To exploit this technique, another substrate integrating transparent electrochemical cell electrodes and microfluidic chambers have to be coupled with the SoG [79,80,81]. Thanks to the developed custom-made connector described in Section 2.2, the ECL technique can also be considered as a future application that could be implemented with the presented system.

### 3.2. Luminescence Detection under Thermal Treatment of the Sample

In this Subsection, we will evaluate the system performances when a thermal treatment of the biological sample is considered. As a first example, we consider the thermochemiluminescence (TCL) process. In this case, the emission of light from the molecule is caused by a chemical reaction induced by heating [82]. In order to perform TCL detection by using the presented system, the simultaneous operation of temperature sensors and photosensors have to be guaranteed.

**Figure 12:**
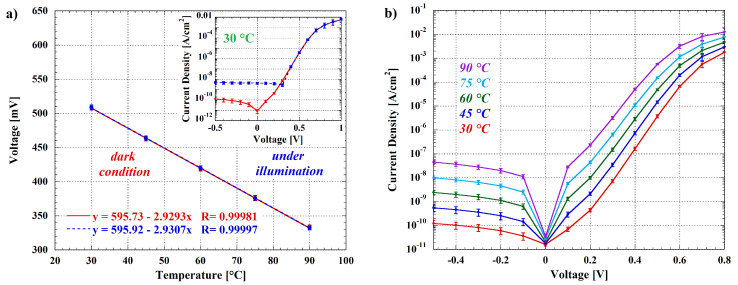
(**a**) Voltage–temperature (VT) characteristics of the fabricated temperature sensors measured in dark condition and in presence of a light intensity of about 1.5 mW/cm^2^; the inset shows the JV graphs measured at 30 °C. Error bars refer to measurements on the six integrated temperature sensors for both VT and JV curves, and standard deviations are less than 3.5 mV and 4 × 10^−4^ A/cm^2^, respectively. (**b**) JV characteristics of the fabricated photosensors measured at different temperatures. Error bars refer to measurements on the eight integrated photosensors, and standard deviations are less than 5 × 10^−3^ A/cm^2^.

As explained in the Section 2, the different top contact of the two sensors allows to measure the temperature of the LoC even in presence of light, as demonstrated in Figure 12a.

In particular, the figure shows the voltage–temperature (VT) characteristic of the fabricated diodes measured with a 50 nA bias current (corresponding to a current density of 5 µA/cm^2^), either in dark condition and in presence of a light intensity of about 1.5 mW/cm^2^, while the inset reports the comparison between the current density–voltage characteristics measured at 30 °C. By looking at the JV curves, it is clear that the presence of light generates a photocurrent that is visible in the reverse voltage bias condition, but which is negligible respect to the bias current, as demonstrated by the agreement of the VT characteristics measured in the two considered conditions.

On the other hand, in order to verify the possibility to use the photosensors during heating, in Figure 12b are reported the JV characteristics measured at different temperatures. As expected, the dark reverse current of the sensors increases exponentially with the temperature, with a consequent raise of the noise related to the light intensity measurement. This exponential law is well known for crystalline silicon diodes as reported in [83], and it applies to a-Si:H diodes too. Even if this matter can represent a limitation for the system usability, it is worth noting that the dark current for reverse voltages near 0 V shows a slow increase, making it feasible to measure the light intensity without major performance losses. Indeed, the dark current measured at 90 °C (a typical value for inducing a thermochemiluminescent process) does not exceed 10 nA/cm^2^ at reverse voltage below 100 mV. This brings a dark current noise equal to 80 fA/(cm^2^√Hz). Following the previous calculations and taking into account that the chemiluminescent spectrum is between 400 and 450 nm, this value leads to an estimation of 270 fA/cm^2^ and 2.4 × 10^6^ photons/(s cm^2^) for the minimum detectable photocurrent and the minimum detectable number of incident photons, respectively. Obviously, the light intensity measurements must be done only when the temperature is stabilized, since during the temperature variations, it is impossible to uncouple the variation of sensor current due to the light from the variation attributable to the temperature.

Another process where it is crucial the luminescent detection and the thermal treatment of the sample is the real-time DNA/RNA amplification process [84,85,86]. Nucleic acid amplification is a fundamental process in molecular biology, since DNA/RNA copies can be used in a large number of medical and forensic applications. It can likewise be used in the identification and detection of infectious diseases [87,88] and for a wide variety of research purposes in the field of molecular genetics, including genetic engineering [89], characterization of species [90], gene therapy [91], and so forth.

Nucleic acid amplification techniques can be subdivided in two main categories, which are polymerase chain reaction-based and isothermal methods [92]. Temperature changes are the main difference between these techniques: in PCR-based ones, a thermal cycler changes the reaction temperatures repeatedly to affect the actions of the temperature-dependent reagents needed for the amplification process [93]; on the other hand, an isothermal amplification reaction occurs at a single temperature [94].

Currently, PCR and derivative technologies are the gold-standard nucleic acid amplification techniques for sensing applications, as they typically have superior sensitivity and specificity [95]. PCR products can theoretically double once during every thermal cycling event; this can provide a more controlled reaction with better specificity but also limits the reaction speed and product yield. PCR also requires precise temperature control and rapid temperature cycling to denature DNA and subsequently anneal and extend short oligonucleotide primers [96]. As this temperature cycling requires additional equipment and time, isothermal nucleic acid amplification technologies fulfill the need for fast and inexpensive molecular detection methods. For these reasons, the isothermal amplification implementation in point-of-care diagnostic devices is greatly simplified, allowing low sample consumption, multiplex DNA analysis, integration, and portable devices realization [97]. Types of isothermal amplification methods include strand-displacement amplification (SDA), rolling-circle amplification (RCA), whole-genome amplification (WGA), loop-mediated isothermal amplification (LAMP), helicase-dependent amplification (HDA), and multiple displacement amplification (MDA), among others.

The detection phase of the target DNA or RNA sample can be done after the amplification step or in real time, depending on the equipment that the chosen platform provides. In our case, the possibility to exploit simultaneously temperature control and optical monitoring allows to perform real time detection by several ways, as demonstrated in [38,98]. In particular, authors have been demonstrated in [98] that, by using a SoG having different geometries for the heater, photosensors and temperature sensors of the LoC presented in this work, it is possible to implement the LAMP technique. In particular, this technique has been optimized to specifically amplify parvovirus B19 DNA and has been coupled with Bioluminescent Assay in Real Time (BART) technology, in order to provide the real-time detection of target DNA. Furthermore, real-time monitoring of MDA has been also performed [38] by exploiting the fluorescence-based method to detect the calf thymus DNA.

**Figure 13:**
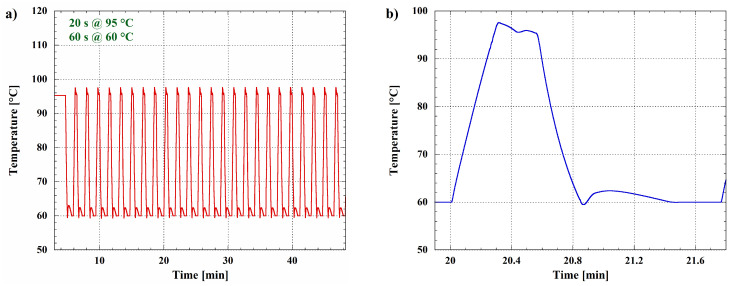
Temperature evolution acquired by the integrated temperature sensor during PCR thermal cycles. (**a**) Graph showing 25 cycles performed at two temperatures; the duration of 95 °C-step is 20 s, while for the 60 °C-step, it is 60 s. (**b**) Magnification showing the temperature evolution during a single PCR cycle.

Depending on the typical application, isothermal amplification methods can have lower sensitivity and/or specificity when compared to PCR. For this reason, the performances of the presented platform have been evaluated to demonstrate the effectiveness also in conducting thermal cycles, in order to implement standard and real-time PCR amplification. Figure 13a shows the temperature evolution during a standard two-temperatures PCR reaction [99], considering 95 °C for the denaturation step and 60 °C for the annealing and the DNA extension.

Figure 13b is a magnification related to a single cycle, where it is possible to appreciate the obtained heating and cooling rates, which are both equal to ∼2.2 °C/s.

**Figure 14:**
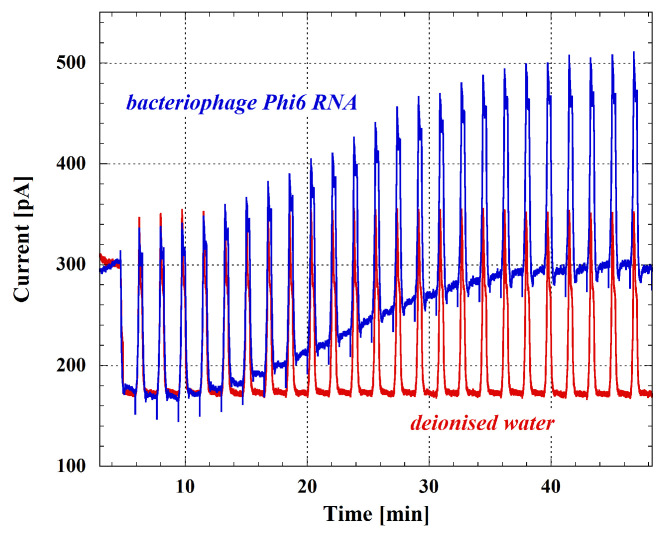
Acquired current during PCR thermal cycles. Red curve refers to a well filled by deionised water, while blue curve shows the current behavior in presence of bacteriophage Phi6 RNA and PCR reaction mixture.

In order to perform real-time monitoring of the nucleic acid amplification, the system has been tested also in presence of the excitation light source for evaluating the acquired photocurrents. In particular, a first step of the experiment was conducted by filling the six wells of the microfluidic chip shown in Figure 10 with deionized water covered by mineral oil to avoid the fluid evaporation. Results are reported as red curve in Figure 14, where it is possible to observe, as expected, the flat behavior of the photocurrent corresponding to the annealing steps. In this case, the current mean value is due to both the value of the sensors dark current at 60 °C and the background light derived from the switched-on excitation LED.

As a second step of the experiment, the deionized water has been replaced by a PCR reaction mixture containing 6 µL of GoTaq PCR master mix (Promega), 1.25 µL of 10 µM forward and reversed primers, 0.3 µL of ruthenium complex 0.1 mM, 0.2 µL of reverse transcriptase (Promega), 2 µL of nuclease free water (Promega), and 1 µL of RNA extracted from bacteriophage Phi6 [100]. This experiment was performed in order to evaluate the system capabilities to monitor the nucleic acid amplification exploiting the fluorescence detection technique. The blue curve of Figure 14 shows the obtained result and, in particular, it is possible to see the increase of the fluorescent signal following a typical sigmoidal-shape trend.

The ability of the proposed platform to perform simultaneously thermal treatments and optical stimulations and monitoring open the doors to several other activities. For example, typical applications that require both heating and optical performances are the cell and bacteria culture processes [101,102,103,104]. Indeed, there is an increasing interest in performing robust and efficient on-chip cell cultures, that can in turn led to more elaborated analysis, like quality evaluation, drug resistance, electropermeabilization, and so on [105,106,107,108,109]. The proposed platform can find its application even in this field, since it allows to perform a biological culture and to provide, at the same time, a functioning analysis of the bioanalyte when stimulated by several external agents, like thermal, chemical, or electrical agents stimuli [110]. Even if the described platform does not claim to be a complete system for cell and bacterial cultures, it certainly represents a good starting point to integrate other kinds of sensors and biocompatible microfluidics for developing on-chip culture system and Organ-on-Chips [20,111,112,113].

## 4. Conclusions

In this work, a multifunctional platform suitable for biochemical and food quality control applications has been presented. The core of the system is an amorphous silicon-based Lab-on-Chip integrating on the same glass substrate thin film light and temperature sensors, a thin film heater suitable for thermal treatment, and an optional thin film interferential filter for fluorescence detection. All the electronics needed to control the LoC and to perform the analysis has also been developed. The presented platform allows to perform the parallelization of the analysis, since on the designed LoC, there are up to six active areas where it is possible to monitor the luminescent signal. Moreover, the whole system has been enclosed in a black metallic box obtaining a portable platform.

System performances have been evaluated as a function of several application tasks. The low dark current of the fabricated photosensors (below 1 pA) allows to reach low detection limits, making the detection and quantification of the target analyte possible by using, for example, chemiluminescent or electrochemiluminescent detection techniques in the visible range (between 400 and 700 nm wavelength). The fluorescent-based detection can also be exploited thanks to the integrated interferential filter, which rejects the excitation source without increasing the distance between the fluorescence site and photosensors. Finally, the simultaneous operation of light and temperature sensors has been demonstrated, allowing several other applications like thermochemiluminescence detection, cell culture processes, and nucleic acid real-time amplification and detection.

The possibility to implement all these kind of analysis makes the presented platform a real multifunctional system suitable for a wide range of application fields.

## Data Availability

Not applicable.

## Abbreviations

The following abbreviations are used in this manuscript:

a-Si:H	hydrogenated amorphous silicon
Al	aluminum
BART	Bioluminescent Assay in Real Time
CL	chemiluminescence
COC	Cyclic Olefin Copolymer
Cr	chromium
ECL	electrochemiluminescence
GUI	Graphical User Interface
HDA	helicase-dependent amplification
ITO	indium-tin oxide
JV	current density–voltage
LAMP	loop-mediated isothermal amplification
LED	light-emitting diode
LoC	Lab-on-Chip
MDA	multiple displacement amplification
PCB	printed circuit board
PCR	polymerase chain reaction
PDMS	Polydimethylsiloxane
PECVD	Plasma-Enhanced Chemical Vapor Deposition
PID	proportional–integral–derivative
RCA	rolling-circle amplification
RIE	Reactive Ion Etching
SDA	strand-displacement amplification
SiO_2_	silicon oxide
SoG	System-on-Glass
TCL	thermochemiluminescence
Ti-W	titanium-tungsten
TiO_2_	titanium oxide
UV	ultraviolet
VT	voltage–temperature
WGA	whole-genome amplification
